# Lichen planus-like eruption resulting from a jellyfish sting: a case report

**DOI:** 10.4076/1752-1947-3-7421

**Published:** 2009-07-23

**Authors:** Sudip Kumar Ghosh, Debabrata Bandyopadhyay, Susmit Haldar

**Affiliations:** 1 Department of Dermatology, Venereology and Leprosy, RG Kar Medical College, 1 Khudiram Bose Sarani, Kolkata, 700004 West Bengal, India; 2 Calcutta Skin Institute, VI M CIT Scheme, Kolkata, 700054 West Bengal, India

## Abstract

**Introduction:**

Contact with a jellyfish can cause a wide variety of conditions, ranging from cutaneous eruption to fatal cardiovascular and respiratory collapse. Cutaneous features can be both acute and chronic. We report a case of persistent lichen planus-like eruption in a young boy after a jellyfish sting, a hitherto unreported occurrence.

**Case presentation:**

A 15-year-old boy presented with multiple lichen planus-like violaceous papules over the lower part of his left thigh on the anterior aspect and also over the patellar region. He had a history of a jellyfish sting over his lower limbs incurred while bathing in the sea four weeks prior to presentation. Histopathology revealed a predominantly perivascular mononuclear cell infiltrate immediately beneath the dermoepidermal junction underneath the hyperplastic epidermis. The lesions significantly subsided with topical corticosteroid application.

**Conclusion:**

This case report demonstrates a new variant of chronic cutaneous change following a jellyfish sting. We report it because of its uniqueness and we believe that physicians should be aware of the possibility of an aquatic animal-induced disease when dealing with lesions with lichen planus-like morphology.

## Introduction

Jellyfish are marine invertebrates found both in the ocean and in fresh water. Jellyfish are categorized into four classes, namely, hydrozoa (Portuguese man-of-war), scyphozoa (true jellyfish), cubozoa (box jellyfish, most toxic) and anthozoa (sea anemones and corals) [1]. Jellyfish are responsible for the most common human envenomations acquired from bathing in the sea. Envenomations usually result in three main types of reactions: immediate allergic, immediate toxic and delayed allergic responses [2]. Fatality can occur due to hypersensitivity or can be induced by the effect of various toxins on the cardiovascular system, respiratory centre or kidneys [3].

Immediate local skin reactions to jellyfish stings at contact sites occur in the form of tenderness, burning and pruritus, which may spread centrally and differ in intensity depending on the species involved. Local soft tissue swellings are common. Erythematous papules and papulo-vesicles, often in a whiplash-like pattern, frequently occur [1]. Ischemic changes distal from localized arterial vasospasm underlying the sting site and thrombophlebitis of the vessel underlying the sting site are also reported. Tender regional lymphadenopathy and distant skin site reactions secondary to a hypersensitive response to the antigenic component of the venom are not uncommon. Uncommon local reactions include angioedema, recurrent reactions, contact dermatitis, and papular urticaria. Delayed or persistent reactions are not uncommon either [3]. Identification of the jellyfish responsible can be made directly by actual viewing of the jellyfish or indirectly with the knowledge of location, time and environmental circumstances of the stinging.

We report a case of persistent lichen planus-like eruption in a young boy as a delayed reaction to a jellyfish sting.

## Case presentation

A 15-year-old boy from rural West Bengal, India, presented with a history of a jellyfish sting on his lower limbs incurred while bathing in the sea in the Bay of Bengal about four weeks earlier. Initially he had an intense burning sensation and swelling of the affected parts along with a skin rash, comprising blisters, redness and superficial ulcerations. There were no systemic symptoms. The initial symptoms subsided with conservative management, including a systemic antibiotic, an analgesic and an antihistamine, but some peculiar asymptomatic skin lesions persisted, compelling him to seek a dermatological consultation. There was no history of any local application on the sites of the jellyfish sting and he reported no past history of any skin disease. In addition, there was no history of a similar illness in the family.

**Figure 1:**
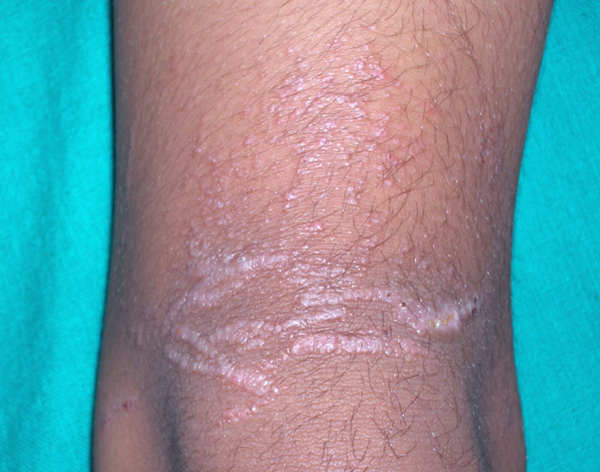
**Lichen planus-like cutaneous lesion: multiple violaceous papules over the lower part of the left anterior thigh and also over the patellar region**. The lesions were discrete as well as confluent in linear fashion in parallel rows mimicking lichen planus.

An examination revealed multiple small papules over the lower part of his left thigh on its anterior aspect and also over the patellar region (Figure 1). The lesions had a distinct violaceous hue and were discrete as well as confluent in a linear fashion in parallel rows. The lesions on his right calf were small papules, grouped in clusters in a linear fashion. Some of these lesions showed crusted erosions. Other areas of his skin were uninvolved and his nails were normal. There was no regional lymphadenopathy or mucosal involvement.

**Figure 2:**
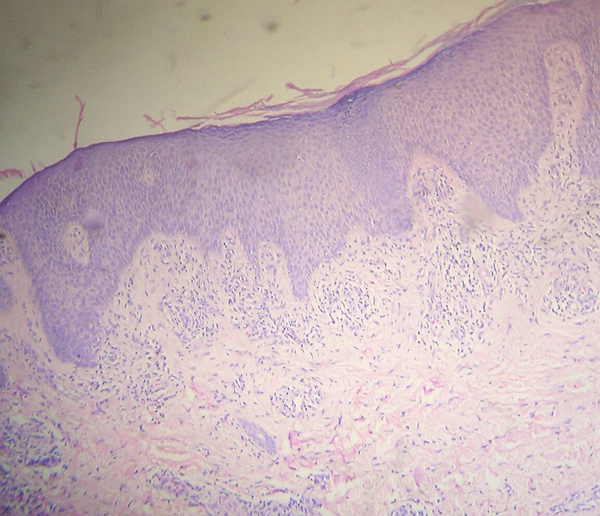
**Histopathology of the cutaneous lesion (original magnification × 100)**. This shows dense mononuclear cell infiltrate immediately beneath the dermo-epidermal junction in perivascular locations, and hyperplastic epidermis with tapering rete ridges. Sparse perivascular infiltrate is also present in the deeper portion of the dermis.

Histopathology with hematoxylin and eosin stain revealed a dense perivascular accumulation of mononuclear cells immediately beneath the dermoepidermal junction underneath an acanthotic epidermis with tapering rete ridges (Figure 2). Focally, a few mononuclear cells were seen infiltrating the basal layer, and vacuolar change of the basal layer was not seen. Perivascular sparse infiltrate was also seen in the deeper portion of the dermis and the subcutis was normal.

The skin lesions significantly subsided with a three-week course of a twice daily topical application of betamethasone dipropionate (0.05%) cream.

## Discussion

Jellyfish stings may be responsible for both acute and chronic forms of cutaneous lesions. While early skin changes following jellyfish stings are acutely inflammatory in nature, long-term or delayed complications of jellyfish dermatitis may occur in the forms of keloids, pigmented striae, and lichenification from persistent rubbing, granuloma, ulceration and necrosis [1]. Rarely, gangrene, fat atrophy, scarring and contractures as well as pigmentary changes can also occur [2,3]. Delayed cutaneous reactions in the form of grouped pink to red-brown coloured papular lesions, which may be distributed in a random fashion or linearly, are described in the literature [4], but we could not find any report of lichen planus-like eruption as a consequence of a jellyfish sting. This cutaneous reaction may represent a persistent delayed hypersensitivity response to an antigenic component of the coelenterate nematocyst. The histopathological features of a persistent lesion caused by a jellyfish sting may reveal a predominantly perivascular and periadnexal lymphohistiocytic infiltrate located primarily in the reticular dermis often admixed with numerous neutrophils and eosinophils [4]. Epidermal changes may include focal spongiosis and exocytosis of lymphocytes [5]. The eruptions usually subside about seven weeks from the time of onset [5].

Despite the absence of pruritus, which is the dominant symptom of lichen planus, our case bore a striking clinical resemblance to lichen planus, an autoimmune inflammatory dermatosis that presents with distinctly violaceous, itchy papules and plaques, often with prominent mucosal involvement. Lichen planus may present with a variety of morphological patterns including a linear variant, and the lesions may leave a prominent postinflammatory hyperpigmentation following resolution. Histopathologically, lichen planus is characterized by hypergranulosis, acanthosis with saw-tooth elongation of rete ridges, a band-like lymphomononuclear cell infiltrate impinging on the dermo-epidermal junction, and vacuolar degenerative changes of the basal cells [6]. The histology of the present case, however, showed a dense, predominantly perivascular accumulation of mononuclear cells in the upper dermis with underlying hyperplastic epidermis. Thus, although there was a clinical similarity, histopathology of the lesions was not typical of lichen planus.

## Conclusion

This case report demonstrates that lichen planus-like lesions could occur as a chronic complication of a jellyfish sting. This case is reported because of its uniqueness and it is felt that dermatologists should be aware of this when dealing with cases of aquatic animal-induced dermatoses.

## Competing interests

The authors declare that they have no competing interests.

## Consent

Written informed consent was obtained from the parent for publication of this case report and any accompanying images. A copy of the written consent is available for review by the Editor-in-Chief of this journal.

## Authors' contributions

SG and SH analysed and interpreted data on the dermatological disease of the patient. DB performed the histological examination of the skin and was a major contributor in writing the manuscript. All authors read and approved the final manuscript.
